# D299T Mutation in CYP76F14 Led to a Decrease in Wine Bouquet Precursor Production in Wine Grape

**DOI:** 10.3390/genes15111478

**Published:** 2024-11-16

**Authors:** Wanhao Liu, Huilin Xiao, Matthew Shi, Meiling Tang, Zhizhong Song

**Affiliations:** 1 Yantai Academy of Agricultural Sciences, Yantai 264000, China; liuwanhao1978@sina.com (W.L.);; 2 Wolfson College, University of Cambridge, Cambridge CB3 9BB, UK; 3 Department of Plant Science, University of Cambridge, Cambridge CB2 3EA, UK

**Keywords:** linalool, cytochrome P450, *in vitro* enzymatic activity, transient expression

## Abstract

Background: Bouquet is a crucial characteristic indicative of wine quality that develops during the aging stage. The cytochrome P450 VvCYP76F14 multi-functionally catalyzes linalool into (*E*)-8-hydroxylinalool, (*E*)-8-oxolinalool, and (*E*)-8-carboxylinalool, which are direct precursors for wine bouquet. Wine bouquet was closely related to VvCYP76F14 activities. Method: The *VvCYP76F14* genes were cloned from five wine grape varieties using a homologous cloning method. The variation in residues of VvCYP76F14s were assessed by multiple alignment of amino acid sequences. Functional studies were implemented by *in vitro* enzyme activity and transient over-expression systems. Results: D299T variation was observed in VvCYP76F14s of ‘Yantai 2-2-08’, ‘Yantai 2-2-19’, and ‘Yantai 2-3-37’ offspring lines, which was correlated with the decreased content of wine bouquet precursors of (*E*)-8-hydroxylinalool, (*E*)-8-oxolinalool, and (*E*)-8-carboxylinalool, respectively. Notably, the key amino acid residue D299 was located at the phase 0 intron positions of *VvCYP76F14* genes isolated from distinct wine grape varieties or offspring lines, respectively. Notably, VvCYP76F14s of the ‘Yantai2-2-08’, ‘Yantai 2-2-19’, and ‘Yantai 2-3-37’ mutant lines exhibited lower *in vitro* enzymatic activities than those of ‘L35’ and ‘Merlot’. In addition, the transient expression of *VvCYP76F14* cloned from ‘L35’ and ‘Merlot’ restored the levels of wine bouquet precursors in berries of three D299T mutant lines, respectively, whereas *VvCYP76F14* cloned from D299T mutant lines failed. Conclusions: D299T variation was observed in three offspring lines and D299T mutation in VvCYP76F14 led to the decrease in wine bouquet precursor contents. VvCYP76F14 was implicated in the regulation of wine bouquet precursors in wine grapes.

## 1. Introduction

Wine aroma is an indispensable sensory indicator of wine quality that further dominates wine consumption [1,2,3,4]. To date, wine aroma largely originates from two sources: primary aromas and secondary aromas (or wine bouquet) [1,4,5]. In detail, primary aromas are generated from flavor compounds that are present in wine grape berries and are embodied in floral, sweet, herbal, and fruity notes. However, wine bouquet is generated through fermentation and the aging process, in which new aromatic compounds are produced after transformations of flavor precursors [4,6,7]. Notably, the primary aromas are inclined to diminish during the aging process, whereas wine bouquet becomes more abundant, defining the unique aromatic profiles of grape wine (*Vitas vinifera*) [3,4,8,9].

Although a vast number of aroma compound have been identified in wine grape, the complex and diverse wine bouquet is attributed to a restricted number of compounds, such as sesquiterpenes, esters, fusel alcohols, and lactones [1,10,11]. Notably, bicyclic monoterpene lactones are involved in the regulation of wine bouquet. However, bicyclic monoterpene lactones are formed from the crucial precursor (*E*)-8-carboxylinalool during the aging stage, but not derived from wine grape berries [12,13].

In wine grape, the biosynthesis of (*E*)-8-carboxylinalool was executed by the Cytochrome P450 enzyme VvCYP76F14 that catalyzes the conversion of linalool into (*E*)-8-hydroxylinalool, (*E*)-8-oxolinalool, and (*E*)-8-carboxyllinalool, which involves hydroxylation, dehydrogenation oxidation, and carboxylation, respectively [8,11,13,14]. In *Arabidopsis*, the redox partner cytochrome P450 reductase (CPR) transfers electrons from the redox partner NADPH to AtCYP76C1 [11,14,15]. However, the physiological function of VvCYP76F14 and the molecular basis or mechanisms underlying the regulation of (*E*)-8-carboxylinalool biosynthesis in wine grapes are essentially rare.

According to previous studies, ‘Neutral’ (low bouquet density), ‘Aromatic’ (middle bouquet density), and ‘Full-Bodied’ (high bouquet density) are standard and classical descriptions of wine grape varieties in terms of wine bouquet [1,8,16,17]. Recently, a close correlation between wine bouquet and VvCYP76F14 enzymatic activity was revealed that was based on the variation in VvCYP76F14 amino acid sequences [8]. In this study, three mutant lines, ‘Yantai 2-2-08’, ‘Yantai 2-219’, and ‘Yantai 2-3-37’, that harbor the D299T variation in their VvCYP76F14s were collected and utilized, and molecular studies of subcellular localization, *in vitro* enzymatic determination, and in vivo transient over-expression of high bouquet type *VvCYP76F14* in berries of D299T mutant lines were conducted to reveal the physiological function of VvCYP76F14 in regulating wine bouquet formation. Favorably, this study helps in investigating the molecular mechanisms and regulations of VvCYP76F14 in wine grapes.

## 2. Results

### 2.1. Difference in Wine Bouquet Precursors Among Different Wine Grape Varieties

Offspring lines of ‘Yantai 2-2-08’, ‘Yantai 2-219’, and ‘Yantai 2-3-37’ are naturally obtained hybrid offsprings of Chardonnay (Neutral type) and Italian Riesling (Neutral type). The determination of wine bouquet precursor contents illustrated that linalool levels in ‘Yantai2-2-08’, ‘Yantai 2-2-19’, ‘Yantai 2-3-37’, ‘Merlot’, and ‘L35’ berries were non-significantly changed (Table 1), whereas (*E*)-8-hydroxylinalool, (*E*)-8-oxolinalool, and (*E*)-8-carboxylinalool levels were significantly different among these wine grape varieties or offspring lines, respectively (Table 1). The highest amounts of three distinct linalool-derivative compounds were found in ‘L35’ berries, followed by ‘Merlot’ berries, and the lowest amounts were observed in ‘Yantai2-2-08’, ‘Yantai 2-2-19’, and ‘Yantai 2-3-37’ berries (Table 1). Interestingly, (*E*)-8-oxolinalool and (*E*)-8-carboxylinalool were not detected in ‘Yantai2-2-08’, ‘Yantai 2-2-19’, or ‘Yantai 2-3-37’ berries. Therefore, we speculate that ‘Yantai 2-2-08’, ‘Yantai 2-2-19’, and ‘Yantai 2-3-37’ are typical Neutral-type wine bouquet varieties.

### 2.2. D299T Variation Was Observed in VvCYP76F14s of Neutral Varieties or Offspring Lines

**Figure 1:**
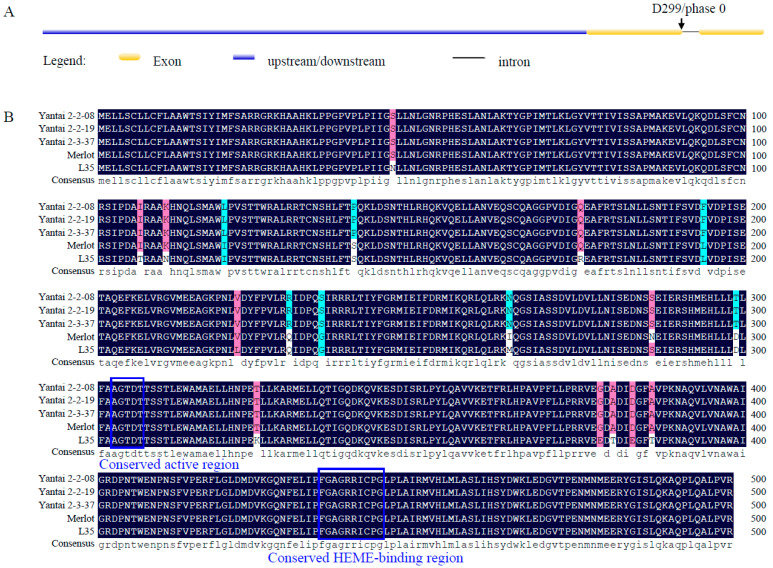
Gene structure and amino acid sequence alignment analysis of VvCYP76F14 proteins. (**A**) Gene structure of *VvCYP76F14*s derived from three mutant lines. (**B**) Acid sequence alignment of VvCYP76F14 proteins.

Variations in VvCYP76F14 amino acid sequences were closely correlated with wine bouquet and VvCYP76F14 enzymatic activity [8]. We isolated the CDSs of *VvCYP76F14*s from ‘Yantai2-2-08’, ‘Yantai 2-2-19’, ‘Yantai 2-3-37’, ‘Merlot’, and ‘L35’ berries. DNA sequencing analysis showed that all five *VvCYP76F14*s possessed an identical gene structure, with an intron inserted at the phase 0 of the D299 residue site (Figure 1A), and encoded a polypeptide consisting of 499 amino acids (Figure 1B). In particular, the AGTDT motif (303–307 site, monooxygenase P450 motif) and FGAGRRICFG motif (435–444 site, HEME-binding region belonging to Class II monooxygenase) were identified in all VvCYP76F14 sequences (Figure 1B).

Although amino acid sequences of ‘Yantai2-2-08’, ‘Yantai 2-2-19’, and ‘Yantai 2-3-37’ VvCYP76F14s were identical, variations were found among these three offspring lines, ‘Merlot’, and ‘L35’ VvCYP76F14s (Figure 1B). Compared to Full-Bodied ‘L35’ VvCYP76F14, 17 site variations (N46S, T107I, N111K, I120L, S140P, R175Q, L194F, L222V, Q230R, G235S, M264N, D299T, K325T, E378G, T380A, E383D, and T386A) were found in ‘Yantai2-2-08’, ‘Yantai 2-2-19’, and ‘Yantai 2-3-37’ Neutral offspring lines, whereas 12 site mutations (N46S, T107I, N111K, R175Q, L222V, M264I, S286N, K325T, E378G, T380A, E383D, and T386A) were identified in Aromatic ‘Merlot’. Compared to Aromatic ‘Merlot’ VvCYP76F14, seven site variations (S140P, L194F, Q230R, G235S, I264N, N286S, and D299T) were found in ‘Yantai2-2-08’, ‘Yantai 2-2-19’, and ‘Yantai 2-3-37’ Neutral offspring lines. Interestingly, D299T variation was only found in VvCYP76F14s of three Neutral lines. However, the expression levels of all *VvCYP76F14* genes were non-significantly changed (Supplementary Figure S1).

### 2.3. Phylogenetic Tree Analysis of Plant CYP76 Family Homologs

**Figure 2:**
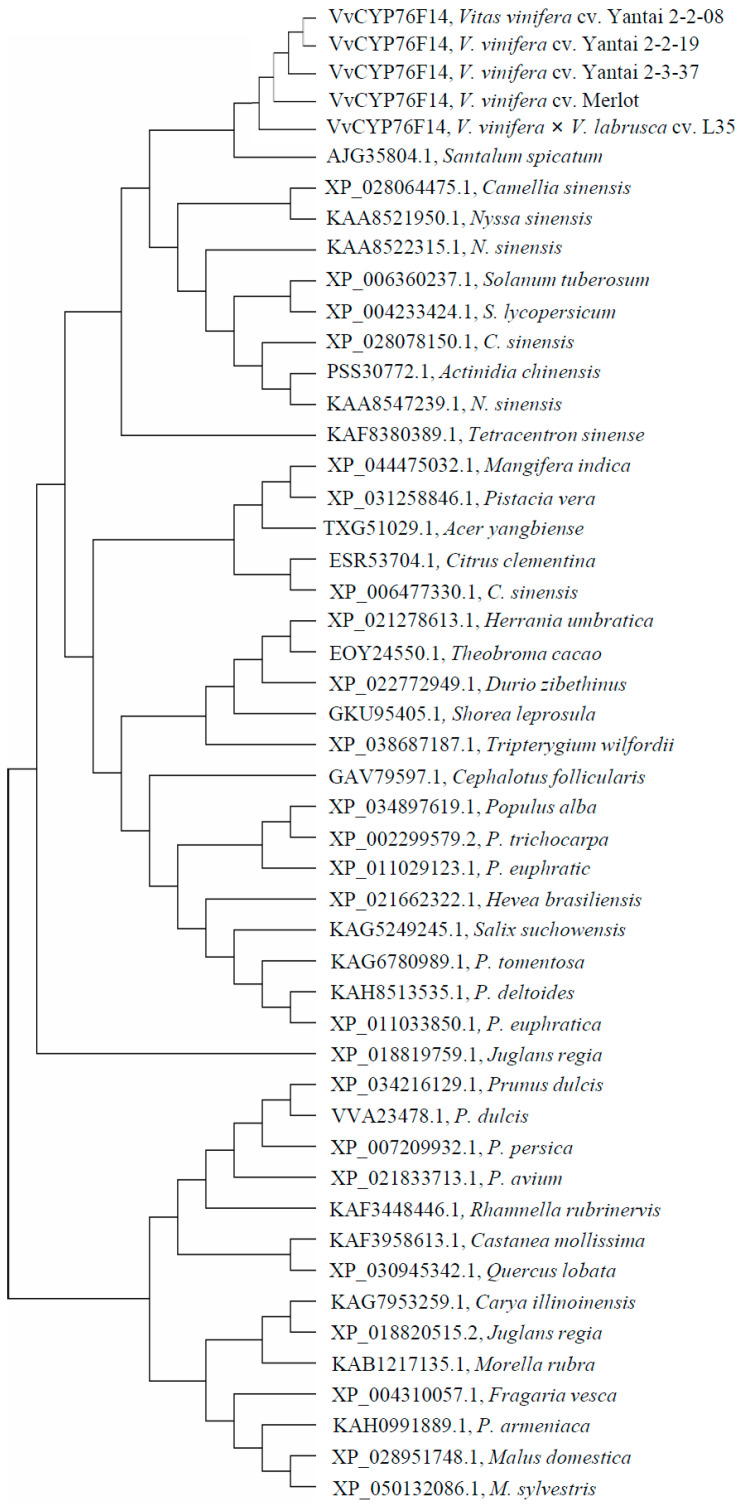
Phylogenetic analysis of plant CYP76 homologs. The phylogenetic tree of plant CYP76 homologs from wine grape and 41 other species was constructed employing the Maximum-Likelihood method in MEGA 13.0 software.

Phylogenetic tree analysis showed that five VvCYP76F14 homologs were closely clustered together, and they exhibited the closest relationship with the corresponding CYP76 homologue from *Santalum spicatum* (Figure 2). Moreover, CYP76 homologs belonging to the same genus or family, such as *Mangifera indica* and *Pistacia vera* of *Anacardiaceae*; *Citrus clementina* and *Citrus sinensis* of *Rutaceae*; *Herrania umbratical*, *Theobroma cacao*, and *Durio zibethinus* of *Malvaceae*; *Populus alba*, *Populus trichocarpa*, *Populus euphratic*, *Salix suchowensis*, *Populus tomentosa*, *Populus deltoides*, and *Populus euphratica* of *Salicaceae*; and *Prunus dulcis*, *Populus persica*, *Populus avium*, *Populus armeniaca*, *Fragaria vesca*, *Malus domestica*, and *Malus sylvestris* of *Rosaceae*, exhibited a closer genetic distance during long-term evolution (Figure 2).

### 2.4. VvCYP76F14 Is Localized in the Endoplasmic Reticulum

**Figure 3:**
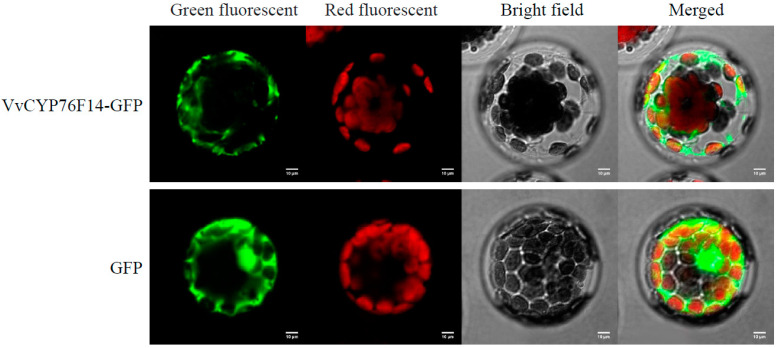
Subcellular localization of VvCYP76F14. The CDS of *VvCYP76F14* derived from ‘Yantai 2-2-08’ and ‘Yantai 2-2-08’ and further cloned into the pBWA(V)HS-ccdb-GLosgfp vector. The GV3101 strain harboring the pBWA(V)HS-CYP76F14-Glosgfp or the empty vector was infiltrated into *Arabidopsis* mesophyll protoplasts. The GFP fluorescence and chloroplast autofluorescence were observed using the excitation/emission wavelengths 470/510 nm and 620/660 nm, respectively. Scale bar = 10 μm.

To investigate the subcellular localization of VvCYP76F14, pBWA(V)HS-ccdb-Glosgfp or pBWA(V)HS-VvCYP76F14-Glosgfp was expressed in *Arabidopsis* mesophyll protoplasts. Confocal determination revealed that the fluorescence of GFP in protoplasts transformed with pBWA(V)HS-ccdb-Glosgfp (empty vector) was observed in various organelles, especially in the cytoplasm, whereas fluorescence of pBWA(V)HS-VvCYP76F14-Glosgfp was determined in the endoplasmic reticulum (ER) (Figure 3), which implies that VvCYP76F14 is a typical ER-localized protein.

### 2.5. Neutral-Type VvCYP76F14s Exhibited Lower Enzymatic Activity In Vitro

**Figure 4:**
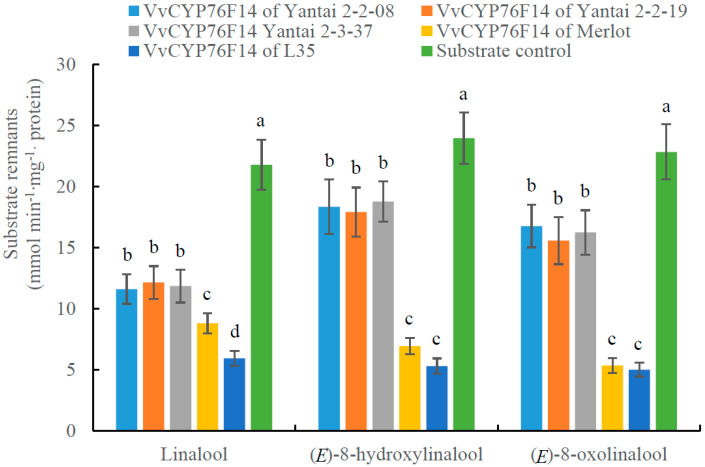
*In vitro* activity analysis of VvCYP76F14 in *Escherichia coli*. The *in vitro* enzymatic activity of VvCYP76F14 was assayed by measuring the amount of remnant substrate. Data are shown as means ± SE (*n* = 3). Letters indicate significant differences among five VvCYP76F14s at a significance level of *p* ≤ 0.05, as determined using ANOVA followed by Fisher’s LSD test.

*In vitro* enzyme activity analysis demonstrated that the catalytic activity of distinct bouquet type VvCYP76F14s in catalyzing linalool, (*E*)-8-hydroxylinalool, and (*E*)-8-oxolinalool was quite different, whereas there were no significant changes in the substrate reduction for the negative control substrate (Figure 4). In detail, the ‘L35’ VvCYP76F14 exhibited lower remnants of linalool and (*E*)-8-hydroxylinalool substrates, compared to ‘Merlot’ and three D299T mutant lines. When taking (*E*)-8-oxolinalool as a substrate, no significant variation was found in the substrate remnants between ‘L35’ and ‘Merlot’ VvCYP76F14s, and this variation was significantly lower than that of the ‘Yantai2-2-08’, ‘Yantai 2-2-19’, and ‘Yantai 2-3-37’ offspring lines (Figure 4). These findings indicate that the Neutral-type ‘Yantai2-2-08’, ‘Yantai 2-2-19’, and ‘Yantai 2-3-37’ mutant lines exhibited lower *in vitro* enzymatic activity of VvCYP76F14.

### 2.6. Transient Expression of the ‘L35’ or ‘Merlot’ VvCYP76F14 Enhanced the Wine Bouquet Precursor Contents in the D299T Substitution Mutant Lines

**Figure 5:**
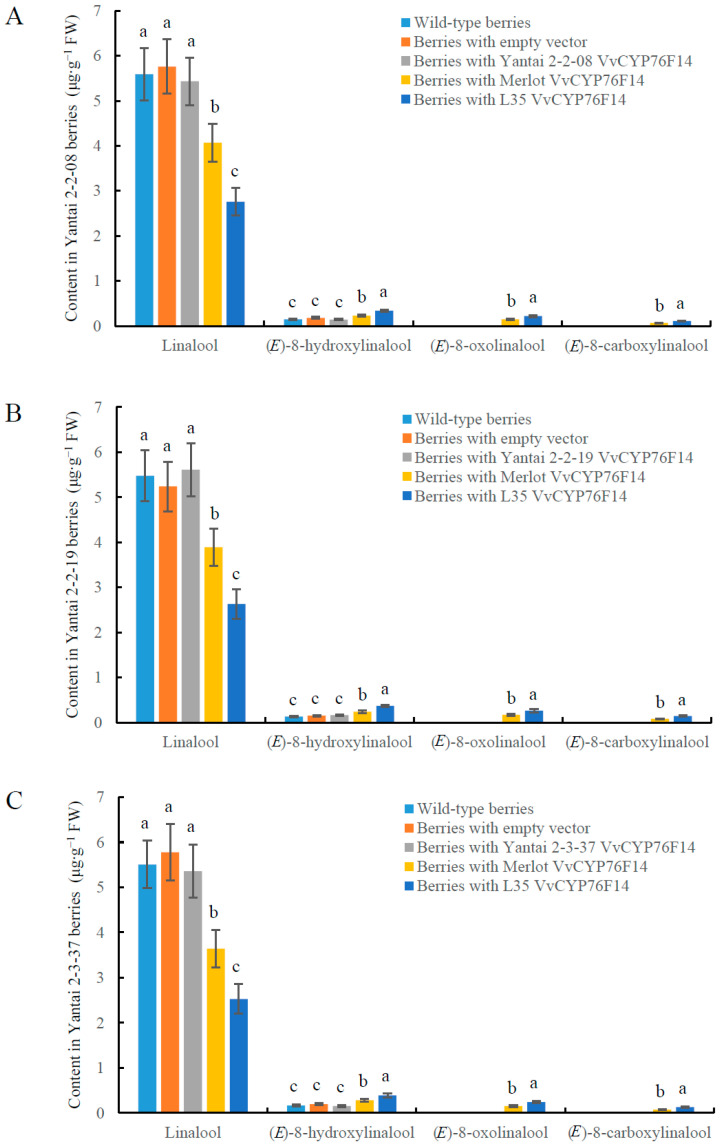
Transient expression of *VvCYP76F14* in berries of D299T mutant lines. The content levels of linalool, (*E*)-8-hydroxylinalool, (*E*)-8-oxolinalool, and (*E*)-8-carboxylinalool in berries of ‘Yantai 2-2-08’ (**A**), ‘Yantai 2-2-19’ (**B**), and ‘Yantai 2-3-37’ (**C**) were assayed by UPLC-MS. Data are shown as means ± SE (*n* = 3). Letters indicate significant differences among five VvCYP76F14s at a significance level of *p* ≤ 0.05, as determined using ANOVA followed by Fisher’s LSD test.

According to the natural screening of over 200 hybrid offspring of Chardonnay and Italian Riesling, the D299T substitution was identified in the ‘Yantai2-2-08’, ‘Yantai 2-2-19’, and ‘Yantai 2-3-37’ mutant lines, which had nearly no levels of (*E*)-8-oxolinalool and (*E*)-8-carboxylinalool (Figure 1B, Table 1). To further unveil the physiological role of D299T substitution on the activity of VvCYP76F14 in vivo, the *VvCYP76F14* CDS isolated from the Full-Bodied variety ‘L35’ or Aromatic variety ‘Merlot’ was transiently over-expressed in ‘Yantai2-2-08’, ‘Yantai 2-2-19’, and ‘Yantai 2-3-37’ berries, respectively. These findings indicated that the contents of (*E*)-8-hydroxylinalool, (*E*)-8-oxolinalool, and (*E*)-8-carboxylinalool in ‘Yantai2-2-08’ berries transformed with ‘L35’ or ‘Merlot’ *VvCYP76F14* were significantly higher than berries transformed with ‘Yantai2-2-08’ *VvCYP76F14*, berries transformed with the pMDC32-HPB empty vector, and the wild-type berries, respectively, and the contents of three distinct linalool-derivative compounds in ‘Yantai2-2-08’ berries transformed with ‘L35’ *VvCYP76F14* were higher than the contents of those transformed with ‘Merlot’ *VvCYP76F14* (Figure 5A). In addition, the level of linalool in ‘Yantai2-2-08’ berries transformed with ‘L35’ or ‘Merlot’ *VvCYP76F14* was significantly lower than that in berries transformed with ‘Yantai2-2-08’ *VvCYP76F14*, berries transformed with the pMDC32-HPB empty vector, and the wild-type berries, respectively. However, the contents of linalool and three linalool-derivative compounds in berries transformed with ‘Yantai 2-2-08’ *VvCYP76F14* were non-significantly changed, compared with berries transformed with the empty vector and the wild-type berries (Figure 5A). Similar findings were observed in the transformed ‘Yantai 2-2-19’ berries (Figure 5B) and ‘Yantai 2-3-37’ berries (Figure 5C).

## 3. Discussion

In wine grapes, the cytochrome P450 protein VvCYP76F14 orderly dominates three catalytic processes (hydroxylation, dehydrogenation, and carboxylation) that catalyze linalool into (*E*)-8-hydroxylinalool, (*E*)-8-oxolinalool, and (*E*)-8-carboxylinalool, respectively [8,11,13,14]. In particular, (*E*)-8-carboxylinalool is the precursor of bicyclic monoterpene lactones that contribute to wine bouquet [8,11,13,14]. However, molecular studies of VvCYP76F14 in regulating wine bouquet precursor formation remain largely unclear.

The active motif signature A(G)G(A)XD(E)T is highly conserved in the Class II monooxygenase domain of the CYP450 family proteins [18,19,20]. In this present study, all five of these *VvCYP76F14s* encoded a 499-amino-acid polypeptide. The AGTDT motif (located at the 303–307 site, a monooxygenase P450 motif) and the FGAGRRICFG motif (located at the 435–444 site, a HEME-binding region belonging to Class II monooxygenase) were both present in these five VvCYP76F14s (Figure 1B), which was consistent with previous reports in *Arabidopsis* [8,18] and yeast [21]. Reactions catalyzed by CYP450s can oxidize substrate and create a metabolic channel on the ER that specifically depends on the typical monooxygenase HEME-binding domain (FGAGRRICFG) [14,22]. Indeed, VvCYP76F14s are ER-pitched proteins (Figure 3), suggesting that VvCYP76F14 may have a significant role in metabolic flux during aroma compound biosynthesis.

In fruit crops, the substitution of key amino acid residues in functional enzymes resulted in aromatic component content variations [23,24]. To date, genetic differences among hybrid offsprings possess the potential to enhance wine bouquet and quality [8,11,25,26]. Picard et al. demonstrated that amino acid sequences of VvCYP76F14s derived from different wine grape varieties were kept the same [27]. In contrast to their findings, amino acid variations were found in these five VvCYP76F14s, which directly reflects their distinct wine bouquet precursor contents in berries and *in vitro* enzymatic activities (Table 1 and Figure 4). These findings imply that VvCYP76F14 could be chosen as a molecular marker to screen amino acid variations in VvCYP76F14 in wine grape offsprings with different wine bouquet types, and further support the proposition that sequence variations in VvCYP76F14s are closely related to wine lactone precursor contents and VvCYP76F14’s enzymatic activities [8].

It is quite interesting that the key amino acid residue D299 was located at the phase 0 intron positions of *VvCYP76F14s* derived from these three D299T mutant lines (Figure 1A). Notably, (*E*)-8-oxolinalool and (*E*)-8-carboxylinalool were hardly detected in D299T mutant lines and the *in vitro* enzymatic activity of these VvCYP76F14s was significantly lower than that of ‘Merlot’ and ‘L35’ (Table 1 and Figure 4). Moreover, over-expression of *VvCYP76F14* from either the Full-Bodied variety ‘L35’ or the Aromatic variety ‘Merlot’ significantly strengthened the production of three distinct linalool-derivative compounds in transgenic berries of all three of these D299T mutant lines (Figure 5). These in vivo observations are consistent with the *in vitro* enzymatic activities of VvCYP76F14s, supporting their role in regulating the formation of wine bouquet precursors. Nonetheless, these D299T mutant lines are unique and innovative materials to reveal the molecular mechanisms and regulatory studies of VvCYP76F14 enzymes in wine grapes.

## 4. Materials and Methods

### 4.1. Chemicals

The primary precursor linalool was purchased from J and K Scientific Co., Ltd. (Shanghai, China). The subsequent precursors of (*E*)-8-hydroxylinalool, (*E*)-8-oxolinalool, and (*E*)-8-carboxylinalool were synthesized and purified by Accela ChemBio Co., Ltd. (Shanghai, China).

### 4.2. Wine Grape

This study was carried out as a collaboration between Yantai Academy of Agricultural Sciences (Yantai, China) and the University of Cambridge (Cambridge, UK) during the years of 2022, 2023, and 2024. According to previous studies [8], ‘Merlot’ is a typical Aromatic-type and ‘L35’ is a traditional Full-Bodied-type wine grape variety. Three unique offspring lines of ‘Yantai 2-2-08’, ‘Yantai 2-2-19’, and ‘Yantai 2-3-37’ are naturally obtained hybrid offsprings of Chardonnay (Neutral type) and Italian Riesling (Neutral type) that carry the same D299T mutation in their VvCYP76F14s, respectively. Wine grape berries with identical maturity stages were harvested from the National Grape Germplasm Repository (Yantai, China), quickly frozen in liquid nitrogen, and then stored at −80 °C in a fridge.

### 4.3. Analysis of Linalool and Linalool-Derived Compounds in Wine Grape Berries

The contents of linalool, (*E*)-8-hydroxylinalool, (*E*)-8-oxolinalool, and (*E*)-8-carboxylinalool in wine grape berries were determined using Ultra-Performance Liquid Chromatography—Mass Spectrometry (UPLC-MS) (Waters, Milford, MA, USA). In particular, linalool and linalool derivatives in wine grape berries were well glycosylated before UPLC-MS analysis, and the acid hydrolysis of wine grape samples was carried out at pH 3. Three biological replicates were performed, each with 40 individual berries.

### 4.4. Isolation and Sequence Analysis of VvCYP76F14s from Different Varieties or Offspring Lines

Total RNA was extracted from ‘Yantai 2-2-08’, ‘Yantai 2-2-19’, ‘Yantai 2-3-37’, ‘Merlot’, and ‘L35’ wine grape berries using a MiniBEST Plant RNA Extraction Kit (TaKaRa, Dalian, China), and DNA contamination was removed using RNase-free Recombinant DNase I (TaKaRa, Dalian, China). The first-strand cDNA was synthesized using a PrimeScript II First Strand cDNA Synthesis Kit (TaKaRa, Dalian, China). The coding sequences (CDSs) of *VvCYP76F14* were amplified using the Prime STAR™ HS DNA polymerase (TaKaRa, Dalian, China) from five wine grape varieties or offspring lines with specific primer pairs (Forward: 5′-ATGGAGTTGTTGAGTTGTCTG-3′; Reverse: 5′-TCAAACCCGTACAGGTAGAGCTTGCAG-3′) [8,13]. The PCR products were sequenced and verified by Shenggong Bioengineering Co., Ltd. (Shanghai, China).

### 4.5. Phylogenetic Tree Analysis

Sequence alignment of VvCYP76f14s from 5 wine grape varieties or offspring lines was analyzed using DNAMAN software 8.0. The phylogenetic tree of plant CYP76 homologs from wine grape and other 41 species was constructed employing the Maximum-Likelihood method in MEGA 13.0 software.

### 4.6. Real-Time Quantitative PCR

The real-time quantitative PCR (qRT-PCR) analysis was carried out via the LightCycler^®^ 480 system (Roche, Inc., Basel, Switzerland) labeled with SYBR Green qPCR Master Mix (TaKaRa, Dalian, China). The primer pair used for qRT-PCR was as follows: F: 5′-TGTTATCCAACACCATAT-3′; R: 5′-TCCCAGCTTCCTCCATCACA-3′ [8].

### 4.7. Subcellular Localization of VvCYP76F14

Subcellular localization of VvCYP76F14 was conducted as described by Peng et al. [28]. The CDS of *VvCYP76F14* (without termination codon) was amplified from ‘Yantai 2-2-08’ and further cloned into the pBWA(V)HS-ccdb-GLosgfp vector (RiORUN, Wuhan, China), using *Bsa*I and *Eco*31I restriction sites, to generate the recombinant vector pBWA(V)HS-CYP76F14-GLosgfp. The GV3101 (pSoup-P19, Weidi Biotechnology Co., Ltd., Shanghai, China) strain harboring the pBWA(V)HS-CYP76F14-Glosgfp or the empty vector was infiltrated into *Arabidopsis* mesophyll protoplasts. Confocal observations were carried out via an Inverted Microscope and a Laser Confocal Scanning Microscope LSM880 (Carl Zeiss, Oberkochen, Germany). The GFP fluorescence and chloroplast autofluorescence were observed using the excitation/emission wavelengths 470/510 nm and 620/660 nm, respectively.

### 4.8. Heterologous Expression of VvCYP76F14 in E. coli

Heterologous expression of *VvCYP76F14* in *E. coli* was conducted as previously described [8]. The CDSs of the *VvCYP76F14*s from ‘Yantai 2-2-08’, ‘Yantai 2-2-19’, ‘Yantai 2-3-37’, ‘Merlot’, and ‘L35’ were synthesized and verified by GenScript Co., Ltd. (Nanjing, China). These confirmed constructs were heterologously expressed in the *E. coli* BL21(DE3) strain. The confirmation of the recombinant proteins was performed using Liquid Chromatography–Tandem Mass Spectrometry/Mass Spectrometry (LC-MS/MS) by Shanghai Bioprofile Technology Co., Ltd. (Shanghai, China).

### 4.9. In Vitro Enzymatic Activity Determination

*In vitro* enzyme activity of VvCYP76F14 was studied according to the description of Peng et al. [8]. The enzyme activities of recombinant VvCYP76F14s from ‘Yantai 2-2-08’, ‘Yantai 2-2-19’, ‘Yantai 2-3-37’, ‘Merlot’, and ‘L35’ were determined using linalool, (*E*)-8-hydroxylinalool, and (*E*)-8-oxolinalool as the substrate, respectively. The *Arabidopsis* CPR (ATR1 [8,14]) was selected as the electron transport redox partner of VvCYP76F14, with the intent to reconstitute the membrane-bound monooxygenase system and minimize the interference from multiple wine grape NADPH CPR homologs. Reactions were carried out at 26 °C for 1 h with agitation, and the substrate reduction and remnants were analyzed using Ultra-Performance Liquid Chromatography–Mass Spectrometry (UPLC-MS) (Waters, Milford, MA, USA) [8,13]. All assays were performed three times, each with 40 individual berries.

### 4.10. Over-Expression of VvCYP76F14 in Berries of D299T Mutant Lines

To evaluate the enzymatic activity of VvCYP76F14 in berries, the CDS of *VvCYP76F14* was isolated from ‘L35’, ‘Merlot’, or D299T mutant lines, respectively, and synthesized by GenScript Co., Ltd. (Nanjing, China). Sequencing-verified *VvCYP76F14* was cloned into the pMDC32-HPB (Addgene: 32078) to generate the recombinant plasmid pMDC32-HPB-*CYP76F14*. The pMDC32-HPB-*VvCYP76F14* over-expression vector and empty vector were each transferred into the GV3101 (WEIDI, Shanghai, China) strain. The GV3101 suspension with an OD_600_ of 0.8 was injected into the veraison-stage berries of ‘Yantai 2-2-08’, ‘Yantai 2-2-19’, ‘Yantai 2-3-37’, ‘Merlot’, and ‘L35’, respectively, in the early morning at three points individually around the diameter of the berries. After injection for three days, the content levels of linalool, (*E*)-8-hydroxylinalool, (*E*)-8-oxolinalool, and (*E*)-8-carboxylinalool in wine grape berries were assayed by UPLC-MS (Waters, Milford, MA, USA). Three biological replicates were performed, each with 40 individual berries.

### 4.11. Statistical Analysis

The significant differences were analyzed in SPSS 13.0 (Armonk, New York, NY, USA) using ANOVA followed by Fisher’s LSD test method.

## 5. Conclusions

D299T variation was observed in VvCYP76F14s of ‘Yantai 2-2-08’, ‘Yantai 2-2-19’, and ‘Yantai 2-3-37’ offspring lines, and was correlated with the decreased content of wine bouquet precursors of (*E*)-8-hydroxylinalool, (*E*)-8-oxolinalool, and (*E*)-8-carboxylinalool and reduced *in vitro* enzymatic activity. Transient expression of *VvCYP76F14* cloned from Full-Bodied- or Aromatic-type varieties restored the levels of wine bouquet precursors in berries of three D299T mutant lines, whereas *VvCYP76F14* cloned from D299T mutant lines failed.

## Figures and Tables

**Table 1:** Determination of wine bouquet precursors from 5 wine grape varieties or offspring lines.

Wine Grape	Linalool (μg·g^−1^ FW)	(*E*)-8-Hydroxylinalool (μg·g^−1^ FW)	(*E*)-8-Oxolinalool (μg·g^−1^ FW)	(*E*)-8-Carboxylinalool (μg·g^−1^ FW)	Wine Bouquet Type
*V. vinifera* cv. Yantai 2-2-08	5.59 ± 0.61 ^a^	0.15 ± 0.021 ^c^	N.D.	N.D.	Neutral
*V. vinifera* cv. Yantai 2-2-19	5.48 ± 0.53 ^a^	0.13 ± 0.018 ^c^	N.D.	N.D.	Neutral
*V. vinifera* cv. Yantai 2-3-37	5.51 ± 0.54 ^a^	0.16 ± 0.022 ^c^	N.D.	N.D.	Neutral
*V. vinifera* cv. Merlot	5.74 ± 0.63 ^a^	1.38 ± 0.15 ^b^	0.89 ± 0.092 ^b^	0.34 ± 0.041 ^b^	Aromatic
*V. vinifera* × *V. labrusca* cv. L35	6.01 ± 0.64 ^a^	3.74 ± 0.41 ^a^	2.23 ± 0.24 ^a^	0.92 ± 0.11 ^a^	Full-Bodied

Notes: FW, fresh weight. Data are shown as the means ± SE (*n* = 3). Letters indicate significant differences at *p* ≤ 0.05, as determined using ANOVA followed by Fisher’s LSD test. N.D., not detectable.

## Data Availability

The original contributions presented in the study are included in the article and Supplementary Materials, further inquiries can be directed to the corresponding author.

## Supplementary Materials

The following supporting information can be downloaded at https://www.mdpi.com/article/10.3390/genes15111478/s1, Figure S1: Expression levels of VvCYP76F14s from five varieties or offspring lines.
