# Supplementary material for: D299T Mutation in CYP76F14 Led to a Decrease in Wine Bouquet Precursor Production in Wine Grape
Source: Genes (Basel). 2024 Nov 16;15(11):1478. doi: 10.3390/genes15111478 (PMC11593623; doi:10.3390/genes15111478)
Supplement: Supplementary file 1 [file genes-15-01478-s001.zip › genes-3309999-supplementary.pdf]

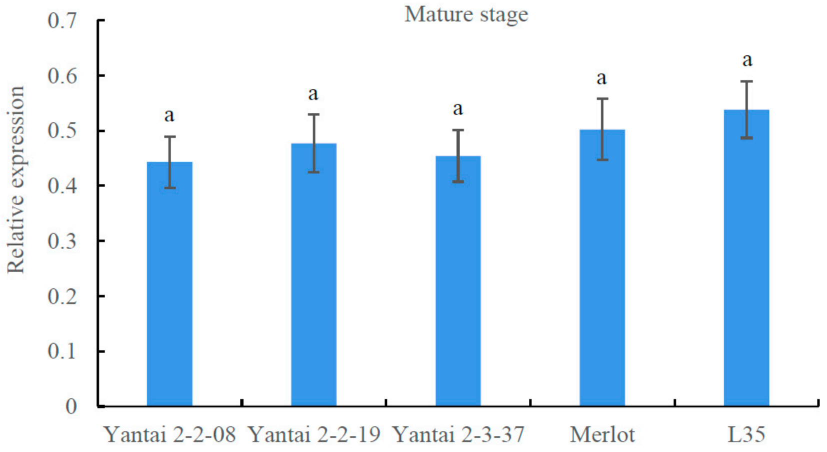

Supplementary Figure S1. Expression levels of *VvCYP76F14s* from five varieties or offspring lines. Data are presented as means ± SEs (n = 3). Letters represent significant differences at a significance level of $P \leq 0.05$, as determined using ANOVA followed by Fisher's LSD test.
